# Reconditioning Emotional Responses With the Break Method: Pilot Quantitative Study

**DOI:** 10.2196/75056

**Published:** 2025-11-07

**Authors:** Boaz Salik, Bizzie Gold, Kira Krier

**Affiliations:** 1 Break Method, Rewire Lab Corporation Sandpoint, ID United States

**Keywords:** mental health intervention, behavior-based therapy, self-guided program, emotional dysregulation, emotional reconditioning

## Abstract

**Background:**

The Break Method is a structured, behavior-based emotional reconditioning program designed to help individuals gain insight into patterns of emotional dysregulation and reprogram behavioral responses rooted in past experiences. Although it has been widely adopted in private and small-group settings, empirical evidence supporting its effectiveness remains limited. With increasing interest in accessible, scalable, and personalized mental health interventions, evaluating the outcomes of such programs is essential for informing future implementation and research.

**Objective:**

This pilot study aimed to evaluate changes in self-reported mental health status before and after participation in the Break Method program. Specifically, we sought to examine (1) overall trends in mental health improvement, (2) associations between specific reasons for joining the program and changes in mental health outcomes, and (3) latent clusters of participant motivations based on symptom profiles.

**Methods:**

Data were collected from 175 unique participants, yielding 195 total survey responses (as 15 participants completed the program more than once). Participants rated their mental health status on a 5-point Likert scale both before and after the program (this was not a validated clinical measure, limiting the interpretability and comparability of results). Descriptive statistics and paired 2-tailed *t* tests were used to assess pre- and postprogram differences in Likert scores. McNemar tests were conducted to compare categorical mental health status (Likert score ≥4 vs <4) before and after participation. Analyses of covariance examined score changes across groups stratified by reported reasons for joining. Multiple correspondence analysis was used to explore latent symptom clusters.

**Results:**

Before program participation, 186 of 195 (95.4%) responses reported Likert scores below 4. Following the program, 157 (80.5%) responses reported scores of 4 or higher. A significant improvement in mental health status was observed (preprogram mean score 2.07 SD 0.82, postprogram mean score 3.92 SD 0.73; *P*<.001). Significant, positive changes were associated with reasons including anxiety (β=0.332, 95% CI 0.073-0.591), obsessive-compulsive disorder (β=0.455, 95% CI 0.061-0.850), and a history of self-harm or suicidal ideation (β=0.511, 95% CI 0.091-0.931). The multiple correspondence analysis identified three clusters of participants based on symptom profiles: (1) low self-image (eg, depression, self-sabotage, and relationship issues); (2) life-development goals (eg, self-discovery and future planning); and (3) obsessive-compulsive disorder–related symptoms. The first cluster was significantly associated with improved mental health outcomes (β=0.348, 95% CI 0.060-0.636).

**Conclusions:**

The Break Method appears to be a promising intervention for improving mental health, particularly among individuals reporting anxiety, low confidence, or a history of self-sabotage. However, due to the single-group, preprogram-postprogram design without a control group, causality cannot be inferred, and these findings should be interpreted as preliminary associations rather than confirmed efficacy. Future studies should incorporate standardized clinical tools, control groups, and longitudinal designs to validate these results and explore long-term outcomes across diverse populations.

## Introduction

Recent studies have linked mental health symptoms, such as depression, to distinct patterns of brain activity observed in nonclinical populations [1]. This growing understanding of the neurobiological and behavioral dimensions of emotional health highlights the importance of interventions that can target maladaptive patterns and promote adaptive coping strategies [2,3]. In response, the Break Method offers a structured, behavior-based approach to emotional reconditioning, combining a data-driven framework with individualized strategies to support emotional regulation and behavioral change.

The Break Method aims to help participants identify and interrupt subconscious patterns that drive automatic, often self-sabotaging behaviors. Although it is not grounded in formal neuroscience protocols, the program draws conceptually from cognitive behavioral principles such as pattern recognition, emotional regulation, and schema restructuring, providing tools for sustainable self-directed change [2,3]. Unlike many traditional interventions that may foster dependency or temporary relief, the Break Method is structured around a defined timeline; self-assessment checkpoints; and a combination of interactive modules, one-on-one sessions, and personalized strategy plans, emphasizing autonomy and long-term skill development. Digital tools have shown potential in reducing stigma and facilitating help-seeking among individuals reluctant to access traditional therapy [4].

This pilot study was conducted to generate foundational insights into the program’s effectiveness and identify patterns of mental health change among participants. Specifically, the study aimed to assess whether improvements in self-reported mental health status varied based on participants’ reasons for enrolling and to explore which psychological profiles might benefit most from this intervention. Understanding these relationships can help optimize the application of the Break Method and inform future research in behavior-based mental health interventions [5].

The main objectives were (1) to evaluate the overall efficacy of the program in improving self-reported mental health symptoms and (2) to determine which specific symptoms or joining reasons are most strongly associated with improvements following participation.

## Methods

### Recruitment

This study recruited participants between fall 2018 and spring 2022. Participants were paying customers of the Break Method who voluntarily enrolled in the program. There were no specific criteria for inclusion based on demographic background, as all individuals who participated in the program during this time frame were invited to complete the survey. Participation in the survey was considered for clients who had opted to be surveyed at program entry. As part of the intake process, participants were screened using a standardized onboarding form, and those reporting active suicidal ideation or crisis-level symptoms were referred to external mental health services and excluded from the program. The survey questionnaire was administered online before and after the intervention. The Break Method in this study was not based on a specific theoretical framework. The questionnaire covered the following topics: early childhood and family relationships, academics, arts and sports, current career and relationship information, and reason for joining the program.

The program is entirely self-paced, with most participants completing it within 4 to 6 months. It includes 4 interactive video modules, 6 one-on-one sessions with a dedicated behavior strategist, a 140-page workbook, and a final strategy session that generates a customized behavior strategy plan ranging from 30 to 100 pages. Participants also had access to a dedicated client support specialist and a community forum for peer support. All activities were conducted through a secure online portal, accessible only via unique usernames and passwords.

Upon program completion, participants were required to complete a survey as part of the exit interview process. The survey was administered via Google Forms and collected participants’ names, email addresses, self-reported mental health status before and after the program, frequency of participation, and reasons for joining. The question about participants’ reasons for joining included 13 response options, allowing participants to select multiple answers: anxiety; depression; relationship issues; poor self-image/low confidence; disordered eating; history of self-harm/suicidal ideation; obsessive-compulsive disorder (OCD)/counting/excessive worry with a calculating nature; parenting issues; addiction; career/workplace issues; self-sabotage/stagnation; future goals/next steps; and general self-discovery. Instructions emphasized the importance of providing honest responses to improve the program’s efficacy.

Demographic data, including age, biological sex, and gender, were also collected but not analyzed in this study. From an initial 286 participants, 20 duplicate responses were removed. Duplicate entries occurred when participants interrupted the response process and resumed it later, resulting in multiple entries for the same individual. Among the remaining participants, those who did not respond to questions about the degree of symptoms (n=91) were excluded, leaving 175 participants in the final analysis.

To ensure transparent and complete reporting, this study followed the CONSORT-EHEALTH (Consolidated Standards of Reporting Trials of Electronic and Mobile Health Applications and Online Telehealth) reporting guidelines for digital interventions.

### Outcomes

Participants’ mental health status was assessed using a 5-point Likert scale for 2 questions in the survey: “How would you rate your symptoms at the start of the Break Method?” “How would you rate your symptoms after completing the Break Method?” Responses ranged from 0 (terrible or debilitating) to 5 (symptoms resolved). Changes in mental health were measured by the difference between the pre- and postprogram Likert scores. We also assessed mental health status by categorizing the Likert scores into 2 groups: 1 for scores ≥4 and 0 for scores <4, applied to both pre- and postprogram scores. We defined a Likert score of ≥4 as *improved* to capture positive changes in self-reported mental health status, as higher scores reflect more favorable outcomes (eg, 4=good and 5=very good). Participants rated their mental health status on a 5-point Likert scale adapted from population health survey frameworks such as the Behavioral Risk Factor Surveillance System [6].

### Ethical Considerations

This study was conducted in adherence to ethical principles governing research involving human participants. All participants provided informed consent via signed agreements facilitated through DocuSign (DocuSign Inc) before taking part in the survey. The consent process ensured that participants were fully informed about the study’s purpose, procedures, and the intended use of their responses. Inclusion or exclusion criteria applied before participation and analysis included excluding individuals actively experiencing suicidal ideation and those requiring crisis-oriented mental health care. Participation in the survey was limited to clients who consented at program entry and completed the program, which may introduce selection bias by excluding dropouts or nonconsenting individuals. However, because the program is voluntary and to respect participants’ choices, this approach was necessary while still allowing us to gather useful information about the program’s effects.

This study qualifies for exemption under 45 CFR 46.104(d)(3), in accordance with the US Department of Health and Human Services (HHS) exemption decision tool (Chart 5), which applies to research involving benign behavioral interventions. The Break Method program, which includes structured emotional skills and self-assessment exercises, constitutes a benign behavioral intervention. Information was collected through self-administered online surveys.

Although some of the information originally recorded (eg, demographic or personal experiences) could have been potentially identifiable, the dataset accessed for this study was fully deidentified before analysis. No identifying variables such as names, contact information, or IP addresses were included in the data available to researchers, and no keys to reidentify participants were retained or accessed. Furthermore, there was no attempt to link survey responses back to any individual.

An institutional review board (IRB) did not conduct a limited review of the study, as permitted under 45 CFR 46.104(d)(3) and illustrated in Chart 5 of the HHS exemption flowchart. A limited review is not necessary when the responses collected do not pose a risk of criminal or civil liability or reputational harm to participants. The topics addressed in the survey, including early childhood and family relationships, academic background, creative and athletic interests, current career and relationship experiences, and reasons for joining the program did not place participants at risk in the event of disclosure.

This research involved a retrospective analysis of data collected between fall 2018 and spring 2022 as part of the Break Method’s internal program assessments. The surveys were administered as part of routine program participation, not for research purposes. No additional data were collected specifically for this study.

In addition, participants exhibiting signs of active suicidal ideation or requiring immediate, crisis-oriented mental health care were excluded from the program at entry, in accordance with the Break Method’s safety protocols. Therefore, the dataset did not include responses from individuals in acute psychological crisis. All data analyzed reflected minimal-risk, retrospective self-reports from participants who completed the full program.

Although this study did not require formal IRB oversight, the research team followed recognized ethical guidelines for studies involving human data. This included respect for participant autonomy, informed consent at point of entry, data confidentiality, and responsible research practices. This exemption classification and ethical posture are consistent with published precedents in peer-reviewed studies, where minimal-risk survey data deidentified and retrospectively analyzed have qualified under 45 CFR 46.104(d)(3) or (d)(2) without formal IRB review.

### Statistical Analysis

Statistical analyses were performed to (1) assess overall changes in symptom severity after participating in the program and (2) examine how changes in symptom severity were associated with the presence of each type of symptom or reasons for joining the program.

The trend of overall changes in mental health symptom severity was estimated using a paired *t* test, comparing the preprogram and postprogram 5-point Likert scale scores for all participants. In addition, the McNemar test, which analyzes paired nominal data in a 2×2 contingency table, was conducted to assess whether there was a significant change in mental health status after the program. This test was applied to a binary variable, where a score ≥4 was coded as 1 and a score ≤4 was coded as 0, both before and after the program.

To explore the second question, the Mann-Whitney *U* test was used to compare preprogram and postprogram means of the Likert scores by the status of each mental health symptom or reason for joining the program. For example, the difference in score changes after the program was compared among participants with and without self-reported responses for anxiety. Additionally, the Cochran-Mantel-Haenszel test [2] was applied to binary variables (ie, 1 for scores ≥4 and 0 for scores <4) of the Likert scale measuring mental health status to assess whether changes in outcomes were significantly associated with each mental health symptom or reason for joining the program.

In our analysis, we modeled change scores as the dependent variable to directly reflect the magnitude of improvement across participant-reported symptom categories. Although analysis of covariance (ANCOVA) is more commonly used to model postintervention scores with baseline scores as covariates, our approach is valid for examining within-subject score changes and between-group differences. We assumed that using change scores was appropriate, given that the actual change from baseline was our outcome of interest and baseline scores appeared similar across participant symptom groups [3]. Nevertheless, future analyses may consider this. Both ANCOVA and change score analyses were conducted to examine pre- and postprogram differences, with ANCOVA controlling for baseline values and change scores providing a complementary perspective. This dual approach allows for a more robust assessment while acknowledging potential limitations in interpretation.

**Figure 1:**
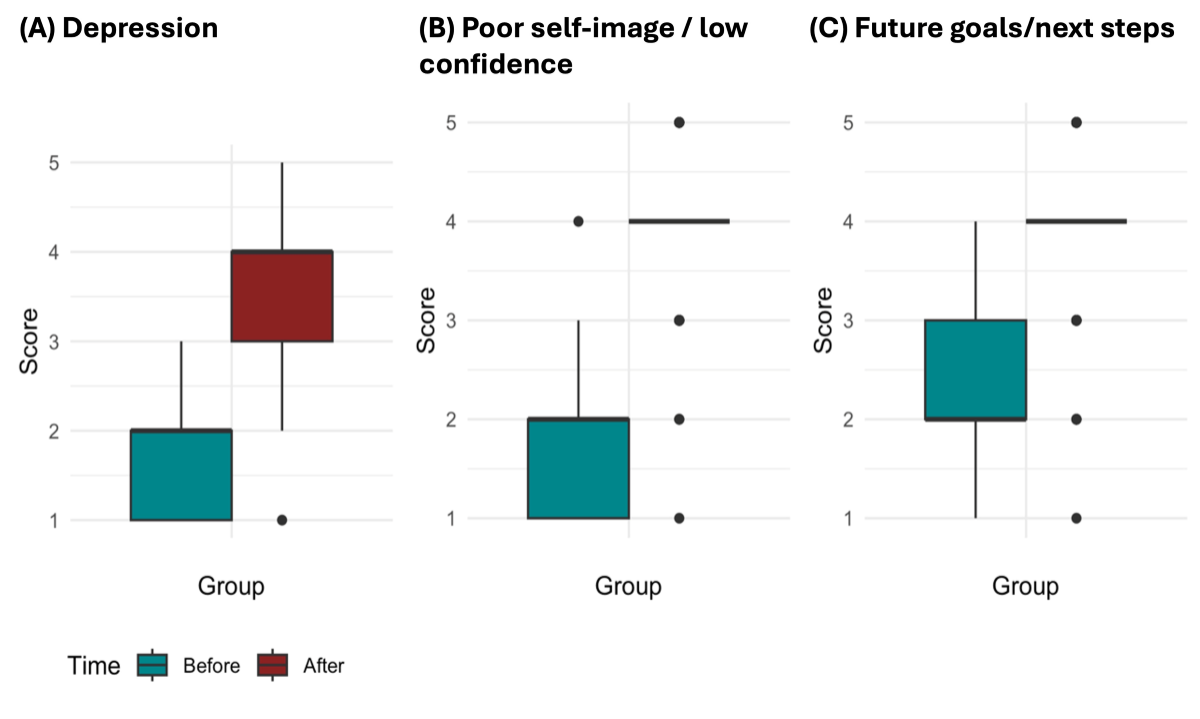
Boxplots of the 5-point Likert score (0: terrible or debilitating; 5: all good) before and after the Break Method program among participants (A) who chose “depression,” (B) “poor self-image/low confidence,” or (C) “future goals/next steps” as their reason for joining.

In addition to the bivariate analysis (Mann-Whitney *U* test and Cochran-Mantel-Haenszel test), a regression approach was conducted to assess the program’s effect on changing mental health status across different factors of mental health symptoms and reasons for joining the program. An ANCOVA, a statistical method combining regression analysis with ANOVA [5], was performed. In this analysis, the change in the Likert score after the program (ie, postprogram score minus preprogram score) was used as the outcome variable, as widely applied in the literature [7]. The ANCOVA model included the presence or absence of a specific reason for joining the program (coded as yes vs no) and controlled for the baseline score (ie, preprogram score). Because baseline scores for mental health status can differ across groups (Figure 1), adjustments for baseline imbalances were applied following recommendations in the literature [8]. This adjustment minimizes bias in estimating the program’s effect by accounting for potential variability in preprogram scores. The ANCOVA was applied to each mental health symptom or reason for joining the program separately.

Multiple responses (eg, depression coexisting with anxiety or self-harm) in the data were highly correlated. To uncover hidden dimensions and clustering, multiple correspondence analysis (MCA), an extension of principal component analysis for categorical variables, was conducted [9]. The MCA decomposes a contingency table (Burt matrix) of categorical variables, identifying patterns and relationships as dimensions in a lower-dimensional space [10]. The number of dimensions was determined using a scree plot, where higher eigenvalues represent a larger share of explained variance. A biplot visualized how reasons for joining the program clustered in 2 dimensions, with principal coordinates calculated based on inertia or eigenvalue criteria [10]. In the biplot, the numbers 1 and 0 next to each variable represent binary outcomes (1=yes; 0=no).

The corresponding coordinates of the individuals, along with the identified dimensions, were used in a generalized estimating equation (GEE) model to evaluate the association of each principal dimension with changes in mental health status after the program. The GEE approach is suitable for repeated measures and clustered data, focusing on population-averaged effects rather than individual-specific effects:



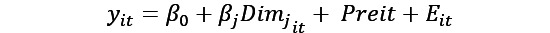



Where y_it_ is the change in the 5-point Likert score for mental health status between pre- and postprogram (ie, postprogram score minus preprogram score) of i-th individual measured at time t, Dim_jit_ is the time-varying variable of the j-th dimension obtained from the MCA (ie, the coordinate on the j-th dimension) of the i-th individual at time t, ε_it_ is the random error term, and β_j_ is the coefficient for the j-th MCA dimension, and Preit is the preprogram baseline Likert score for the mental health status. The model assumed a constant correlation between repeated measures for an individual. The β_0_ represents how the principal MCA dimension affects the outcome (ie, changes in mental health status). The GEE model was applied to principal coordinates derived from the MCA, which summarized correlated categorical variables (eg, overlapping symptoms). Importantly, only unique participant responses were included in the analysis after removing duplicates, so no repeated measures over time were involved. The GEE was therefore used to account for potential correlation within the dimensions identified by the MCA, rather than repeated measurements per individual. To make the GEE approach more accessible, we briefly describe it as a method that accounts for correlations between repeated measurements within participants. The model estimates average changes across the sample while adjusting for within-subject dependencies.

In this study, participants reported a variety of co-occurring mental health symptoms, making it impossible to define mutually exclusive symptom groups. The MCA is a data reduction technique suitable for categorical data to address this issue by identifying latent patterns of co-occurring symptoms. The MCA allows clustering participants based on the similarity of their reported symptoms, creating distinct dimensions. This approach enabled us to examine which participant clusters showed greater changes in mental health outcomes after the program, offering a more interpretable result than symptom-specific comparisons, which can be vulnerable to type 1 error. We then used GEE to examine how the latent dimensions identified through the MCA were associated with temporal changes in mental health status. The GEE provided interpretable statistical estimates, including *P* values and 95% CIs, to assess meaningful differences in mental health changes across participant clusters.

Although the combined MCA+GEE approach is methodologically innovative, the modest sample size (n=175) may limit the stability of the model. This introduces a potential risk of overfitting that should be considered when interpreting the findings.

Multiple statistical tests were performed in this study (eg, paired *t* test, McNemar, Mann-Whitney *U*, ANCOVA, MCA, and GEE), which may increase the risk of type 1 error. A formal correction (eg, Bonferroni or false discovery rate) was not applied, as such methods can be overly conservative in moderate sample sizes like ours and may obscure potentially meaningful exploratory findings. Nonetheless, results should be interpreted with caution given this limitation.

The statistical analyses were conducted in R Software (version 4.2.2; R Foundation for Statistical Computing).

## Results

We used data from a total of 175 participants, of whom 15 responded to the survey 2 or more times during the study period, resulting in 195 unique responses. Table 1 illustrates the frequency of responses for joining the Break Method. The most frequently chosen reason among participants was “self-sabotage/stagnation” (149/175, 85.1%), followed by “future goals/next steps” (147/175, 84%) and “general self-discovery” (147/175, 84%). Other commonly selected responses included “anxiety” (116/175, 66.3%), “relationship issues” (120/175, 68.6%), and “poor self-image/low confidence” (105/175, 60%). The least chosen responses were “history of self-harm/suicidal ideation” (19/175, 10.9%) and “OCD” (22/175, 12.6%).

**Table 1:** Reasons for joining the program. At recruitment and before the program start, participants completed a survey on their reasons for joining. Multiple responses were allowed. Labels reflect the original survey wording (n=175).

Reason	Frequency, n (%)
Anxiety	116 (66.3)
Depression	72 (41.1)
Relationship issues	120 (68.6)
Poor self/image/low confidence	105 (60)
Disordered eating	47 (26.9)
History of self-harm/suicidal ideation	19 (10.9)
OCD^a^/counting/excessive worry with a calculating nature	22 (12.6)
Parenting issues	55 (31.4)
Addiction	28 (16)
Career/workplace issues	67 (38.3)
Self-sabotage/stagnation	149 (85.1)
Future goals/next steps	147 (84)
General self-discovery	147 (84)

^a^OCD: obsessive-compulsive disorder.

Among the 195 responses, 186 (95.4%) respondents reported Likert scores lesser than 4 before the program (Table 2). After the program, 157 (80.5%) responses reported Likert scores of 4 or higher. The paired *t* test revealed significant increases in the 5-point Likert scale after program participation (preprogram mean score 2.07, SD 0.82, postprogram mean score 3.92, SD 0.73; *P*<.001). The McNemar test for the 2×2 table comparing mental health status (eg, ≥4 vs <4 Likert score) before and after the program also indicated a significant difference in the frequency of mental health status (*P*<.001).

**Table 2:** Frequency (%) of the 5-point Likert score for mental health status before and after the program participation (n=195).

Score	Before the program, n (%)	After the program, n (%)
1	48 (24.6)	1 (0.5)
2	95 (48.7)	8 (4.1)
3	43 (22.1)	29 (14.9)
4	8 (4.1)	125 (64.1)
5	1 (0.5)	32 (16.4)

The average of preprogram and postprogram 5-point Likert scores stratified by each reason for joining the program is shown in Table 3.

**Table 3:** Preprogram and postprogram Likert score means, stratified by the joining reasons.

Reason			Before the program, mean (SD)		After the program, mean (SD)		*P* value^a^	
**Anxiety**								<.001
	With	2.42 (0.68)		3.94 (0.73)				
	Without	1.84 (0.87)		3.91 (0.73)				
**Depression**								.15
	With	1.69 (0.66)		3.72 (0.77)				
	Without	2.29 (0.82)		4.03 (0.68)				
**Relationship issues**								.07
	With	1.95 (0.85)		3.86 (0.78)				
	Without	2.27 (0.74)		4.01 (0.63)				
**Poor self-image/low confidence**								.01
	With	1.84 (0.73)		3.87 (0.71)				
	Without	2.34 (0.85)		3.98 (0.75)				
**Disordered eating**								.14
	With	1.79 (0.76)		3.81 (0.73)				
	Without	2.16 (0.83)		3.95 (0.72)				
**Self-sabotage/stagnation**								.03
	With	1.93 (0.75)		3.86 (0.71)				
	Without	2.54 (0.86)		4.11 (0.74)				
**General self-discovery**								.55
	With	2.11 (0.82)		3.99 (0.72)				
	Without	1.96 (0.80)		3.71 (0.73)				
**History of self-harm/suicidal ideation**								.01
	With	1.47 (0.62)		3.89 (0.63)				
	Without	2.14 (0.82)		3.92 (0.74)				
**OCD^b^/counting/excessive worry with a calculating nature**								.01
	With	1.55 (0.69)		3.91 (0.96)				
	Without	2.14 (0.81)		3.92 (0.70)				
**Parenting issues**								.25
	With	2.00 (0.84)		3.98 (0.59)				
	Without	2.10 (0.81)		3.89 (0.78)				
**Career/workplace issues**								.11
	With	1.85 (0.79)		3.84 (0.82)				
	Without	2.19 (0.71)		3.96 (0.67)				
**Future goals/next steps**								.12
	With	2.13 (0.80)		3.95 (0.74)				
	Without	1.90 (0.87)		3.83 (0.69)				
**Addiction**								.05
	With	1.68 (0.68)		3.89 (0.65)				
	Without	2.14 (0.82)		3.92 (0.74)				

^a^The *P* value is based on the Mann-Whitney *U* test comparing the changes in the preprogram and postprogram scores between the groups with and without the specified reason for joining.

^b^OCD: obsessive-compulsive disorder.

Using the ANCOVA, we compared mean changes in Likert scores (postprogram score minus preprogram score) across groups stratified by the presence of each reason for joining the program (Table 4). The β coefficient, indicating the score changes after the program, was highest for “history of self-harm/suicidal ideation” (β=0.511, 95% CI 0.091-0.931), followed by “OCD/counting/excessive worry with a calculating nature” (β=0.455, 95% CI 0.061-0.850). Positive and significant changes in scores were also associated with “anxiety” (β=0.332, 95% CI 0.073-0.591). The smallest absolute score change was observed for “parenting issues” (β=0.132, 95% CI 0.020-0.244).

**Table 4:** Results of the analysis of covariancea.

Variable	β (95% CI)	*P* value
Anxiety	0.332 (0.073 to 0.591)	.01
Depression	0.107 (−0.158 to 0.371)	.43
Poor self-image/low confidence	0.203 (−0.053 to 0.459)	.12
Disordered eating	0.149 (−0.146 to 0.444)	.32
Self-sabotage/stagnation	0.020 (−0.290 to 0.329)	.90
General self-discovery	0.213 (−0.079 to 0.505)	.15
History of Self-harm/suicidal ideation	0.511 (0.091 to 0.931)	.02
OCD^b^/counting/excessive worry with a calculating nature	0.455 (0.061 to 0.850)	.03
Parenting issues	0.155 (−0.125 to 0.434)	.28
Career/workplace issues	0.093 (−0.174 to 0.360)	.50
Future goals/next steps	−0.106 (−0.399 to 0.186)	.48
Relationship issues	0.132 (−0.126 to 0.391)	.32
Addiction	0.297 (−0.062 to 0.655)	.11

^a^The model was adjusted for the baseline Likert score differences.

^b^OCD: obsessive-compulsive disorder.

**Figure 2:**
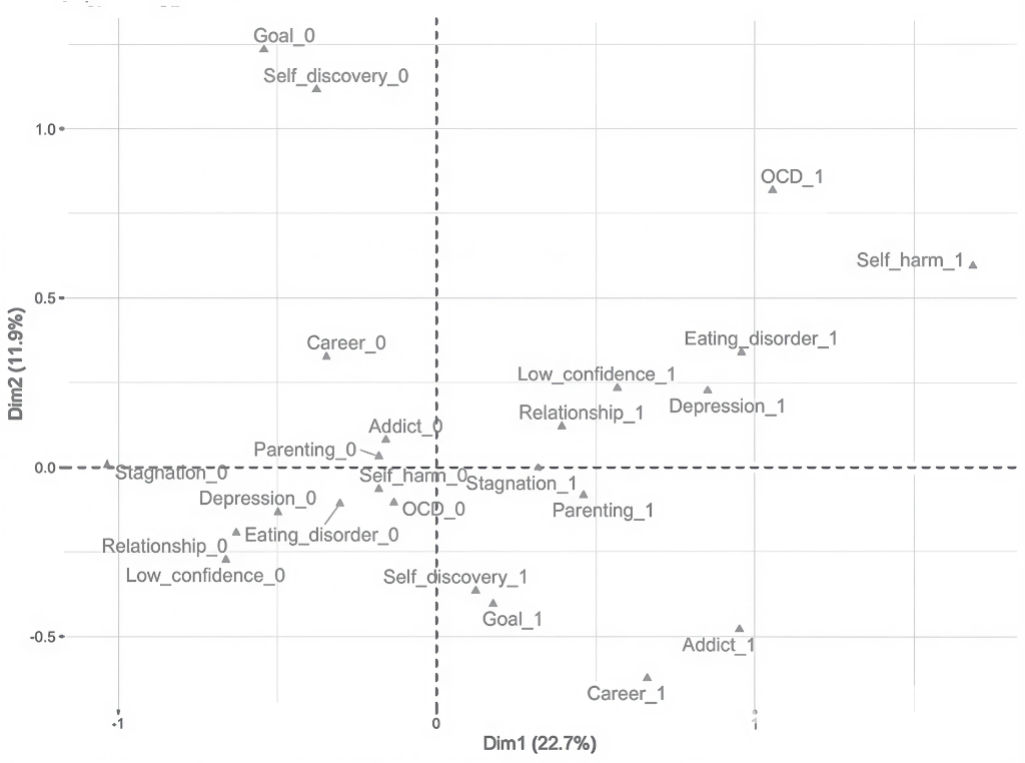
Biplot of the multiple correspondence analysis. OCD: obsessive-compulsive disorder.

The estimated eigenvalues indicated how much variance each dimension explained in the MCA. Dimension 1 accounted for 22.7% of the data variance, while dimensions 2 and 3 accounted for 11.9% and 10.0%, respectively. The biplot of the MCA (Figure 2) provided a graphical representation of relationships between the different categories of reasons for joining the Break Method in the reduced multidimensional space. By examining dimensions 1 and 2, we observed a cluster of participants exhibiting shared characteristics, including depression, low confidence, relationship issues, and self-sabotage or stagnation. Another distinct cluster comprised participants focused on future goals, self-discovery, and career or workplace challenges. Finally, a third category appeared to consist of participants displaying OCD-related symptoms.

The coefficients for the MCA latent dimensions were examined in relation to changes in the 5-point Likert scores for symptoms upon completing the program. Dimension 1 (ie, “low self-image”) was significantly and positively associated with higher increases in Likert scores (β=0.348, 95% CI 0.060-0.636), indicating significant effects among participants who joined the program due to depression, low confidence, relationship issues, or self-sabotage or stagnation. Dimension 2 (eg, self-discovery and future goals) indicated a positive but nonsignificant effect (β=0.168, 95% CI −0.205 to 0.540). Dimension 3 (ie, “OCD”) was not significantly associated with changes in Likert scores (β=−0.031, 95% CI −0.423 to 0.362).

## Discussion

### Principal Findings

This study recruited participants for the Break Method and assessed its impact on their mental health, focusing on the participants’ characteristics as determined by their reasons for joining the program. The results indicated that several factors, including anxiety, OCD, and a history of self-harm or suicidal ideation, were significantly associated with positive changes in Likert scores for mental health status after program participation.

Our analyses of temporal changes in Likert scale mental health status are subject to potential type I error; that is, observed changes among participants with a specific symptom may not solely reflect the intervention’s effect on that symptom, but could also be influenced by co-occurring symptoms. Although this limitation affects the precision of symptom-specific effects, our data collection captured all self-reported symptoms, making it infeasible to define mutually exclusive case and control groups. Despite this limitation, the findings offer valuable insight into temporal patterns of mental health change in individuals reporting specific symptoms. The primary aim of this pilot study was to identify overall trends in mental health status over time. Future studies should use larger samples and validated symptom-specific measures to improve specificity and causal inference.

Our analysis revealed 2 to 3 distinct clusters for participants’ reasons for joining the Break Method. Participants who joined the program for reasons related to depression, low confidence, relationship issues, and self-sabotage or stagnation tended to form a cluster, suggesting a shared pattern of challenges and motivations. Another distinct cluster consisted of participants seeking support for future goals, self-discovery, or career or workplace challenges, highlighting a different set of motivations. Finally, participants associated with OCD-related issues appeared as a third, less interconnected cluster, emphasizing the variability in outcomes for this group.

The findings from our MCA may indicate specific benefits of the Break Method program in medical relevance for psychological symptoms and behavioral patterns. The first MCA dimension clustered symptoms that suggest that individuals in this group may benefit most from emotion-focused reconditioning interventions. The second dimension aligned well with motivational or career coaching approaches. The third dimension may be best addressed through brain pattern mapping and structured self-inquiry exercises. However, because participants were not grouped exclusively by any one dimension or symptom, this study could not assess the program’s effectiveness for each specific cluster, warranting more rigorous future research. This study with a single-group, preprogram-postprogram design lacked a control group, limiting causal inference on intervention effectiveness. However, a randomized controlled trial was not ethically or practically feasible. Designed as a pilot, this study aimed to assess feasibility and potential changes in mental health outcomes rather than establish causality. Despite this limitation, within-subject comparisons offer valuable insight into temporal trends and possible intervention effects. Future research should include appropriate control groups to validate effectiveness. All participants who enrolled completed the program and exit surveys. Therefore, no dropout data were available. This should be considered when interpreting the results.

The Break Method’s use of coaching and digital modules for emotional reconditioning aligns with recent studies [11,12] that support improved delivery of care through technology-based mental health programs that combine digital tools with coaching approaches. Despite its theoretical basis, a key limitation of this study is the lack of published randomized controlled trials evaluating the efficacy. To advance the evidence base, future research should incorporate rigorous designs, such as randomized trials or longitudinal studies, using validated clinical and neuropsychological outcome measures. Evaluating the Break program as both a standalone treatment and an adjunct to existing therapies could also clarify its role in improving mental health care. As engagement is a critical determinant of success in digital interventions [13], understanding user motivation within Break Method remains essential.

After participating in the Break Method program, participants reported increased scores on a 5-point Likert scale, indicating improved mental health status. Notably, greater improvements were observed among those who joined due to depression, low confidence, relationship issues, or self-sabotage and stagnation compared with others. However, the interpretation of these results is limited by the heterogeneity of reported symptoms, as participants experienced a range of conditions including depression and OCD, making it difficult to attribute improvements to specific symptom domains. Although some participants reported reductions in OCD-related symptoms, the Break Method is not a disorder-specific intervention and was not designed to directly treat OCD. These reported changes may instead reflect broader improvements in anxiety and maladaptive thought-behavior patterns. Practically, this highlights potential utility as an adjunctive tool for clients with mixed symptom presentations, while established disorder-specific treatments remain essential. Nonetheless, several limitations must be considered when interpreting these findings.

Despite its promising findings, this study has limitations that should be acknowledged. One key limitation is that while demographic data (eg, age and gender) were collected, they were not analyzed in relation to program outcomes. This omission restricts the ability to examine potential correlations between demographic factors and the program’s effectiveness. Although demographic data were collected, analyses focused on overall symptom change to maintain clarity and statistical power, as the sample size limited subgroup comparisons. This growing field lacks relevant studies, limiting comparison. Our study adds to emerging evidence and underscores the need for further research. Additionally, the study used a 5-point Likert scale to assess the mental health status, rather than using validated clinical tools such as the Patient Health Questionnaire-9 [14]. The use of a nonstandardized measure introduces potential variability in the assessment of mental health improvements. This restricts the interpretability and comparability of outcomes with other studies and may introduce measurement variability. Therefore, key outcomes, such as depression and anxiety, may not have been fully captured. The tool was also subject to recall bias, social desirability bias, and measurement error due to variability in how participants interpreted the scale. Additionally, self-reported symptom “resolution” (score=5) is subjective and may reflect participants’ expectations or social desirability bias rather than true clinical improvement, further limiting interpretation of the findings. However, the use of a full-length, validated instrument was not feasible given the study’s design and its primary aim to evaluate overall changes in mental health following the intervention. Future studies should incorporate validated measures to more accurately assess mental health outcomes and intervention effectiveness.

Another limitation is that all participants were paying clients of the Break Method, which may have influenced motivation or expectations and could have affected self-reported outcomes. This factor may modestly limit generalizability to broader populations. Furthermore, reliance on self-reported data may have contributed to response bias, as participants’ perceptions, expectations, or tendencies toward social desirability could have influenced their reported improvements. Additionally, our analysis used the MCA to group participants based on similarities in mental health symptoms, as exclusively compatible groups for each symptom could not be clearly defined. Given the exploratory nature of the MCA and the limited sample size, including additional covariates risked overfitting and reduced interpretability. Future studies with larger samples and appropriate control groups should investigate the influence of these demographic factors on intervention effectiveness. The study excluded individuals reporting active suicidal ideation or requiring crisis-oriented care, so the findings may not be fully generalized to the high-risk population with active suicidal ideation. However, the results remain informative for participants without acute risk. Finally, the absence of a long-form questionnaire limited the ability to capture more nuanced insights into participants’ mental health status before and after the program. Fully automated digital interventions have demonstrated measurable improvements in mental well-being across diverse populations [15].

In our results of the ANCOVA for each mental health symptom, we did not find significant score changes after the program among those who reported depression or parenting issues. It is important to note that these findings may be vulnerable to type I error. Even if the program had a positive effect on depression, the overall change may have been obscured by the presence of co-occurring symptoms that were not directly addressed by the intervention. Otherwise, depression is more chronic and recurrent, and there is evidence suggesting that depression often requires a longer intervention duration compared with other symptoms such as anxiety, due to its clinical course and risk of relapse [16]. Additionally, parenting issues are complex and usually require longer-term tailored interventions [17]. This evidence suggests that a longitudinal study with a long-term intervention would be needed to observe meaningful improvements in these symptoms, warranting future studies. Consistent with findings from self-guided digital mental health programs [18], participants in our study reported significant postprogram improvements. Scalable digital mental health programs have shown promise in expanding access to care, particularly in low-resource contexts [19]. Future work could also explore age-specific adaptations of self-guided digital interventions, as shown in adolescent populations [20].

### Conclusions

This study suggests preliminary associations between participation in the Break Method and improvements in self-reported mental health status, particularly among individuals struggling with anxiety, low confidence, and self-sabotage. These findings highlight the importance of structured, behavior-based interventions in addressing emotional dysregulation and fostering initial mental health improvements. By identifying key participant clusters, this research suggests that personalized intervention strategies could enhance program effectiveness and inform future refinements of the Break Method.

Although the study demonstrates improvements in self-reported outcomes, the single-group preprogram-postprogram design without a control group prevents causal conclusions. The observed changes may be influenced by confounding factors or natural symptom fluctuation, highlighting the need for controlled trials to rigorously assess the Break Method’s effectiveness.

This study underscores the need for further research to explore the long-term efficacy of the Break Method, its applicability across diverse populations, and its potential integration into broader mental health care systems. These findings serve as a foundation for developing more tailored, scalable interventions that can be adapted across different mental health treatment settings. Future studies should ideally include independent evaluations conducted by researchers not affiliated with the Break Method to strengthen the objectivity and credibility of the findings.

## Abbreviations

ANCOVA: analysis of covariance
CONSORT-EHEALTH: Consolidated Standards of Reporting Trials of Electronic and Mobile Health Applications and Online Telehealth
GEE: generalized estimating equation
IRB: institutional review board
MCA: multiple correspondence analysis
OCD: obsessive-compulsive disorder

## Data Availability

The datasets generated or analyzed during this study are not publicly available to maintain participant confidentiality and to remain in compliance with ethical standards governing the use of sensitive human data. Deidentified data may be made available from the corresponding author upon reasonable request and with appropriate ethical approval.
